# Supplementary material for: Tunable Bessel beam two-photon fluorescence microscopy for high-speed volumetric imaging of brain dynamics
Source: eLife. 2026 Jun 19;15:RP110228. doi: 10.7554/eLife.110228 (PMC13282114; doi:10.7554/eLife.110228)
Supplement: Peer review history

# Editor report: eLife Assessment

**DOI:** 10.7554/eLife.110228.2.sa0

**Authors:** Brice Bathellier

**Affiliations:** Centre National de la Recherche Scientifique France

**Keywords:** Convincing, Important

This **important** study substantially advances the imaging toolbox available to neuroscientists by presenting a tunable Bessel (tBessel-TPFM) platform that enables high-speed volumetric two-photon imaging. The evidence supporting the novel methodology is **convincing**, with rigorous benchmarking and demonstrations of a wide range of neuroimaging applications covering vascular dynamics, neurovascular coupling, optogenetic perturbation, and microglial responses. The work will be of broad interest to neuroscientists and imaging system tool developers.

# Referee report: Reviewer #1 (Public review)

**DOI:** 10.7554/eLife.110228.2.sa1

Summary:

This manuscript presents a tunable Bessel-beam two-photon fluorescence microscopy (tBessel-TPFM) platform that enables high-speed volumetric imaging with stable axial focus. The work is technically strong and broadly significant, as it substantially improves the flexibility and practicality of Bessel-beam-based two-photon microscopy. The demonstrations are generally strong and bridge a wide range of neuroimaging applications, namely vascular dynamics, neurovascular coupling, optogenetic perturbation, and microglial responses. These convincingly show that the approach enables biological measurements that are difficult or impractical with existing methods.

The evidence supporting the technical and biological claims is generally strong. The optical design is carefully motivated, clearly described, and validated through a combination of simulations and experimental characterization. The biological applications are diverse and well chosen to highlight the strengths of the proposed method, and the data are of high quality, with appropriate controls and comparative measurements where relevant.

Strengths:

(1) The optical innovation addresses a well-recognized limitation of existing Bessel-TPFM implementations, namely axial focus drift during tuning, and does so using a relatively simple, light-efficient, and cost-effective design.

(2) The manuscript provides convincing experimental evidence for this being a versatile platform to map flow dynamics across diverse vessel sizes and orientations in both healthy and pathological states.

(3) Biological demonstrations are comprehensive and span multiple domains such as hemodynamics, neurovascular coupling, and neuroimmune responses.

(4) Quantitative analyses of blood flow across vessel sizes and orientations, including kilohertz line scanning, are particularly compelling and clearly beyond the reach of standard Gaussian TPFM.

(5) Particular advantages are that higher blood slow speeds become measurable up to 23mm/sec (20x more than conventional frame scanning), and that simultaneous (Bessel-)imaging and (Gaussian-)perturbation are possible because of the stable axial focus.

Weaknesses:

(1) At present, the paper does not properly position the new Bessel-beam method against previous work, and fails to compare it to alternative fast volumetric imaging methods without Bessel beams.

(2) The cost-effectiveness of the proposed method is not well described or supported by evidence; it would be useful to include more detail or remove this claim.

(3) Some biological conclusions, e.g., regarding novel features of microglial dynamics (i.e., the observed two-wave responses and coordinated extension-retraction), are based on relatively limited sample size and would benefit from clearer discussion of variability across animals and fields of view.

(4) The use of neural network-based denoising for microglial imaging is reasonable but introduces potential concerns about trustworthiness; additional clarification of validation or failure modes would strengthen confidence in these results.

To conclude, most of the authors' claims are well supported by the data. The central conclusion, namely that tBessel-TPFM provides tunable volumetric imaging enabling experiments not feasible with existing two-photon approaches, is justified. Some biological interpretations would benefit from a more cautious framing, but they do not undermine the main technical and methodological contributions of the study. This is a strong and technically rigorous manuscript that makes a substantial methodological advance with clear relevance to neuroscience and intravital imaging. Minor clarifications and a slightly more measured discussion of certain biological findings are recommended.

# Referee report: Reviewer #2 (Public review)

**DOI:** 10.7554/eLife.110228.2.sa2

Summary:

The authors describe a tunable Bessel beam two-photon microscope (tBessel-TPFM) designed to overcome a common limitation of Bessel-based volumetric imaging: axial shifts of the effective focus during Bessel beam parameter tuning. Their optical design allows independent control of axial beam length and resolution while keeping the axial center fixed. This is extensively validated through simulations and experiments.

Strengths:

A major strength of the work is the breadth of validation combined with the level of technical detail provided. The authors carefully characterize the optical performance of the system and clearly explain the design choices and underlying derivations, which will make it easier for others to understand and implement. The authors demonstrate the utility of the method across several in vivo applications, including neurovascular imaging, blood flow measurements, optogenetic stimulation, and microglial dynamics.

Weaknesses:

In the in vivo demonstrations, the authors employ different Bessel beam configurations across experiments, but the beam parameters are not dynamically tuned during live imaging. A video example showing continuous or interactive tuning of the Bessel beam within a single in vivo imaging sequence would further highlight the practical advantages of this platform and strengthen the case for its potential applications. In addition, while excitation powers are reported, the manuscript does not place these values in the broader context of known photodamage thresholds for two-photon microscopy, which would be helpful to the readers. Denoising/image restoration are applied in one of the in vivo examples, but it is unclear why this step was used specifically for this dataset and whether it was necessary to achieve adequate SNR or primarily included as an additional demonstration.

# Referee report: Reviewer #3 (Public review)

**DOI:** 10.7554/eLife.110228.2.sa3

Summary:

The manuscript presents an elegant and cost-effective approach for generating a tunable Bessel beam on a conventional two-photon microscope. The authors assemble a compact optical module comprising three axicons and a series of lenses that permits rapid adjustment of both lateral resolution and axial extent without modifying the focal plane. This flexibility enables the system to be readily adapted to a variety of biological preparations. As a proof of concept, the authors employ the device to record blood flow velocities in cortical microcapillaries, arterioles, and venules, thereby directly visualizing vasodilatation and vasoconstriction dynamics and permitting quantitative analysis of neurovascular coupling across cortical layers in awake mice.

The authors demonstrate that the tunability of the Bessel beam can be exploited to match the numerical aperture to the vessel type: a high NA configuration, albeit slower scan, is optimal for resolving flow in capillaries, whereas a low NA setting provides faster acquisition suitable for arterioles and venules. By implementing a one-dimensional line scan with the Bessel beam, they achieve an imaging speed that is twentyfold faster than conventional frame-by-frame scanning, which proves sufficient to capture hemodynamic transients before and after an induced ischemic stroke.

In addition to pure observation, the authors integrate a co-propagating Gaussian line to the system, allowing simultaneous imaging and photostimulation within the same focal plane. This capability addresses a common limitation of other Bessel beam implementations, in which the observation and perturbation planes often become misaligned when the Bessel beam is altered. The manuscript also emphasizes the advantage of Bessel beam excitation for calcium imaging after a perturbation, because it captures neuronal activity in planes both above and below the nominal focal plane, signals that would be missed with a standard Gaussian focus. Finally, the authors apply the technique to investigate the neuroimmune response following targeted microglial ablation; they report that adjacent microglia extend processes toward the injury site while retracting processes in the opposite direction.

Overall, the work offers a technically straightforward yet powerful extension to existing two-photon platforms, providing high-speed, volumetric imaging and stimulation capabilities that are well-suited to a broad range of neurovascular and neuroimmune studies. The experimental validation is quite thorough, and the presented data convincingly illustrates the benefits of the approach.

Strengths:

The authors present a truly clever and inexpensive optical module that can be integrated into almost any two-photon microscope, providing a tunable Bessel beam with a minimal modification of the existing system. The experimental data and accompanying quantitative analysis convincingly demonstrate that the system can reveal physiological events, such as capillary flow, calcium transients across multiple axial planes, and microglial process dynamics, that are difficult or impossible to capture with a conventional Gaussian beam. The breadth of experiments chosen for the manuscript illustrates the practical utility of the device and supports the authors' conclusions that it extends the functional repertoire of standard two-photon microscopy.

Weaknesses:

The manuscript would benefit from a more detailed contextualisation of the claimed speed advantage. Although the authors mention other techniques in the introduction, they do not provide any direct comparison with other state-of-the-art high-speed two-photon approaches such as light beads microscopy (Demas et al., Nat. Methods 2021), temporal multiplexing schemes (Weisenburger et al., Cell 2019), or random access microscopy (Villette et al., Cell 2019). A brief comparison of imaging speed, spatial resolution, and instrumental complexity would enable readers to assess the relative merits of the present method.

A second limitation that warrants discussion is the inherent trade off between volumetric coverage and image specificity. Because the Bessel beam excites fluorescence throughout an extended axial range, the detector inevitably integrates signal from a three dimensional volume into a two dimensional image. In densely labelled tissue, this can lead to significant signal crosstalk, reducing contrast and complicating quantitative interpretation. A brief analysis of how labeling density affects the fidelity of flow or calcium measurements, or suggestions for mitigating crosstalk (e.g., computational deconvolution, adaptive excitation shaping, or combinatorial sparse labeling), would broaden the applicability of the technique.

# Author comment: Author response

**DOI:** 10.7554/eLife.110228.2.sa4

**Authors:** Tian-Ming Fu, Mengyang Jacky Li, Jinghui Wang, Mikolaj Walczak, Yuqing Qiu, Colleen Russell, Miroslaw Janowski, Piotr Walczak, Yajie Liang

**Affiliations:** Princeton University Princeton United States; Princeton University Princeton United States; University of Maryland School of Medicine Baltimore United States; University of Maryland School of Medicine Baltimore United States; Princeton University Princeton United States; University of Maryland School of Medicine Baltimore United States; University of Maryland School of Medicine Baltimore United States; University of Maryland School of Medicine Baltimore United States; University of Maryland, Baltimore Baltimore United States

**Public Reviews:**

**Reviewer #1 (Public review):**

This manuscript presents a tunable Bessel-beam two-photon fluorescence microscopy (tBessel-TPFM) platform that enables high-speed volumetric imaging with stable axial focus. The work is technically strong and broadly significant, as it substantially improves the flexibility and practicality of Bessel-beam-based two-photon microscopy. The demonstrations are generally strong and bridge a wide range of neuroimaging applications, namely vascular dynamics, neurovascular coupling, optogenetic perturbation, and microglial responses. These convincingly show that the approach enables biological measurements that are difficult or impractical with existing methods.

The evidence supporting the technical and biological claims is generally strong. The optical design is carefully motivated, clearly described, and validated through a combination of simulations and experimental characterization. The biological applications are diverse and well chosen to highlight the strengths of the proposed method, and the data are of high quality, with appropriate controls and comparative measurements where relevant.

Strengths:

(1) The optical innovation addresses a well-recognized limitation of existing Bessel-TPFM implementations, namely axial focus drift during tuning, and does so using a relatively simple, light-efficient, and cost-effective design.

(2) The manuscript provides convincing experimental evidence for this being a versatile platform to map flow dynamics across diverse vessel sizes and orientations in both healthy and pathological states.

(3) Biological demonstrations are comprehensive and span multiple domains such as hemodynamics, neurovascular coupling, and neuroimmune responses.

(4) Quantitative analyses of blood flow across vessel sizes and orientations, including kilohertz line scanning, are particularly compelling and clearly beyond the reach of standard Gaussian TPFM.

(5) Particular advantages are that higher blood slow speeds become measurable up to 23mm/sec (20x more than conventional frame scanning), and that simultaneous (Bessel-)imaging and (Gaussian-)perturbation are possible because of the stable axial focus.

We thank the reviewer for this thoughtful and encouraging evaluation of our work. We are particularly grateful for the recognition of both the technical rigor and the broad applicability of the tBessel-TPFM platform, as well as the assessment that our approach enables biological measurements that are difficult or impractical with existing methods. We appreciate the reviewer’s detailed summary of the strengths of the manuscript, including the identification of axial focus drift as a major limitation in prior Bessel-TPFM implementations, and the value of our center-stable, light-efficient, and accessible solution. We thank the reviewer for the encouraging comment that our biological demonstrations to be compelling and well supported by quantitative analysis.

Weaknesses:

(1) At present, the paper does not properly position the new Bessel-beam method against previous work, and fails to compare it to alternative fast volumetric imaging methods without Bessel beams.

We thank the reviewer for this important point. We agree that a more explicit comparison with existing fast volumetric imaging methods helps clarify the unique advantages of our system. Alternative fast volumetric imaging methods without Bessel beams include remote focusing (Sofroniew et al., 2016), acousto-optic deflectors (AOD) (Villette et al., 2019), piezoelectric objective stages (Göbel and Helmchen, 2007), tunable acoustic gradient lenses (TAG lens) (Huang et al., 2019), electrically tunable lenses (ETLs) (Grewe et al., 2011; Yang et al., 2018), and light beads microscopy (Demas et al., 2021). These methods have each enabled important forms of rapid volumetric imaging, but they differ in their speed, resolution, axial range, and optical complexity. For example, remote focusing can provide rapid axial refocusing while preserving high-resolution imaging but has limited defocus range and requires a carefully aligned relay system and aberration control to maintain image quality. AOD-based approaches enable fast random-access sampling, but introduce optical and calibration complexity associated with dispersion, and suffer light loss with limited diffractive efficiency. Piezoelectric objective scanning is comparatively simple and broadly accessible, but its mechanical inertia limits volume rate and can introduce artifacts during rapid or large axial motion. TAG lenses and ETLs provide compact non-mechanical axial scanning, but pose challenges on aberration control and synchronization. Light-beads microscopy achieves high volumetric throughput by near-simultaneously sampling multiple axial positions, but faces intrinsic compromise among axial coverage, number of sampling planes, and lateral sampling density, which limit lateral resolution when imaging over large depth ranges.

Previous Bessel-beam TPFM approaches address some of these limitations by converting volumetric imaging into two-dimensional scanning with an axially extended focus. However, many existing implementations either rely on a fixed Bessel beam profile, which limits the ability to adapt spatial resolution and axial coverage to different biological applications, or use spatial light modulators, which provide tunability but introduce higher cost, increased optical complexity, reduced light efficiency, and sequential rather than simultaneous multi-wavelength operation. Other axicon or lens based tunable Bessel approaches have also been reported, but these designs generally introduce axial displacement of the Bessel focus during tuning.

In contrast, our tBessel-TPFM design provides full tunability comparable with SLM based methods, maintaining a stable axial beam center, at the same time low cost, easy to implement, intrinsically high light efficiency and support simultaneous multi-color imaging. Therefore, tBessel-TPFM provides a unique solution for applications where axial projection is acceptable and where high-speed volumetric monitoring, tunable axial coverage, motion robustness, optical simplicity, and compatibility with simultaneous perturbation are valuable.

(2) The cost-effectiveness of the proposed method is not well described or supported by evidence; it would be useful to include more detail or remove this claim.

We thank the reviewer for requesting clarification and supporting evidence regarding the cost-effectiveness of our method. We now provide a detailed cost breakdown of the tBessel module. Briefly, the module consists of three axicons, three lenses, and one iris that together enable independent control of the NA and ΔNA of the generated Bessel beam. Based on the specified components, the three axicons (AX252B and AX255B, Thorlabs) cost $635 each, the three lenses (AC254-125-B×2 and AC254-150-B, Thorlabs) cost $110 each, and the iris (SM2D25D, Thorlabs) costs $105, resulting in a total system cost of approximately $2,340. For comparison, spatial light modulator (SLM)-based implementations that offer comparable tunability typically require an SLM module costing on the order of $20,000 USD, in addition to more complex optical alignment and reduced optical efficiency.

(3) Some biological conclusions, e.g., regarding novel features of microglial dynamics (i.e., the observed two-wave responses and coordinated extension-retraction), are based on relatively limited sample size and would benefit from clearer discussion of variability across animals and fields of view.

We thank the reviewer for this important comment regarding the limited sample size of the microglial dynamics study. We agree that a more comprehensive assessment across animals would be required to establish the generality of these biological findings. In the current study, our intent is not to draw broad biological conclusions, but rather to report observations enabled by the tBessel-TPFM platform. As noted in the manuscript, we have deliberately used descriptive language (e.g., “two distinct waves of process extension were observed” “process dynamics revealed…” and “advancing processes displayed…”) to avoid over claim of the biological findings beyond the data presented.

(4) The use of neural network-based denoising for microglial imaging is reasonable but introduces potential concerns about trustworthiness; additional clarification of validation or failure modes would strengthen confidence in these results.

We thank the reviewer for raising this important point regarding the reliability of neural network-based denoising. We agree that additional validation and discussion of potential failure modes are essential to build confidence in these results. To assess the fidelity of the CARE-denoised data, we performed several additional analyses (Author response image 1). First, we compared normalized raw and denoised images averaged over 10 frames. The difference between the two images was spatially uniform and primarily reflected residual noise present in the raw data, rather than structured discrepancies (Author response image 1a). As expected, brighter features like microglial somata exhibited smaller differences due to their intrinsically higher signal-to-noise ratio, whereas weaker processes showed larger noise-related differences. Second, we extended this comparison across the full time-lapse sequence by applying consistent color mapping to both raw and denoised videos and computing frame-by-frame difference maps. These analyses show that the observed differences are consistent with noise suppression, without introducing coherent structural features or altering the apparent microglial dynamics (Author response image 1b).

**Author response image 1.** Validation of CARE-based denoising for microglial imaging. (a) Comparison of 10-frame averaged normalized raw (left), CARE-denoised (middle), and their pixel-wise difference (right) images. The second row shows a zoomed-in view of the boxed region. (b) Color-coded time-lapse projections over a 10-min imaging session for the raw (left) and CARE-denoised (middle) data, along with their pixel-wise difference (right).

To conclude, most of the authors' claims are well supported by the data. The central conclusion, namely that tBessel-TPFM provides tunable volumetric imaging enabling experiments not feasible with existing two-photon approaches, is justified. Some biological interpretations would benefit from a more cautious framing, but they do not undermine the main technical and methodological contributions of the study. This is a strong and technically rigorous manuscript that makes a substantial methodological advance with clear relevance to neuroscience and intravital imaging. Minor clarifications and a slightly more measured discussion of certain biological findings are recommended.

We thank the reviewer for this thoughtful and encouraging summary of our work. We greatly appreciate the recognition that tBessel-TPFM provides a meaningful methodological advance and enables volumetric imaging experiments that are difficult or impractical with existing two-photon approaches.

**Reviewer #2 (Public review):**

The authors describe a tunable Bessel beam two-photon microscope (tBessel-TPFM) designed to overcome a common limitation of Bessel-based volumetric imaging: axial shifts of the effective focus during Bessel beam parameter tuning. Their optical design allows independent control of axial beam length and resolution while keeping the axial center fixed. This is extensively validated through simulations and experiments.

Strengths:

A major strength of the work is the breadth of validation combined with the level of technical detail provided. The authors carefully characterize the optical performance of the system and clearly explain the design choices and underlying derivations, which will make it easier for others to understand and implement. The authors demonstrate the utility of the method across several in vivo applications, including neurovascular imaging, blood flow measurements, optogenetic stimulation, and microglial dynamics.

We thank the reviewer for their thoughtful and encouraging comments. We greatly appreciate the recognition of the technical rigor, breadth of validation, and clarity of explanation presented in our work.

Weaknesses:

In the in vivo demonstrations, the authors employ different Bessel beam configurations across experiments, but the beam parameters are not dynamically tuned during live imaging. A video example showing continuous or interactive tuning of the Bessel beam within a single in vivo imaging sequence would further highlight the practical advantages of this platform and strengthen the case for its potential applications.

We thank the reviewer for their suggestion. While we agree that continuous or interactive tuning of the Bessel beam during imaging would further highlight the practical flexibility of the platform, and changing the Bessel beam parameters during imaging session is feasible in our tBessel-TPFM implementation, for the in vivo applications presented in this manuscript, dynamic tuning during the actual recording is generally not required. In practice, the Bessel beam parameters are selected before data acquisition based on the biological target, desired axial coverage, spatial resolution, and acceptable level of projection overlap.

In addition, while excitation powers are reported, the manuscript does not place these values in the broader context of known photodamage thresholds for two-photon microscopy, which would be helpful to the readers.

We thank the reviewer for bringing up this important point. It is known that multiphoton imaging relies on relatively high illumination power, which causes brain heating and thus photodamage. Previous studies have reported that continuous illumination with a 920-nm laser beam at 0.8 NA over 1000s results in a peak temperature increase of ~1.73 °C/100 mW in the brain, with power above 300 mW observed to cause cellular damage. Power levels below 250 mW were considered to be safe for long-term imaging. (Podgorski and Ranganathan, 2016) In our experiments, the measured post-objective powers range from 20 mW to 149 mW, which are well below the established safe threshold.

Denoising/image restoration are applied in one of the in vivo examples, but it is unclear why this step was used specifically for this dataset and whether it was necessary to achieve adequate SNR or primarily included as an additional demonstration.

We thank the reviewer for requesting clarification on the usage of the CARE denoising model. The CARE-based denoising was applied only in Figure 5, the microglial imaging example, and was primarily included as an additional demonstration of how neural network–based image restoration can be used to enhance low-SNR volumetric datasets acquired with tBessel-TPFM. All other images and analyses in the manuscript were performed on raw data without any denoising. To assess the reliability of the CARE denoising method, we further compared raw and denoised data using 10-frame averages and color-mapped the full 10-minute time-lapse video, both showed minimal differences (Response Fig 1). These analyses confirm that the CARE denoising model did not introduce structural artifacts or affect the biological dynamics observations in our dataset.

**Reviewer #3 (Public review):**

The manuscript presents an elegant and cost-effective approach for generating a tunable Bessel beam on a conventional two-photon microscope. The authors assemble a compact optical module comprising three axicons and a series of lenses that permits rapid adjustment of both lateral resolution and axial extent without modifying the focal plane. This flexibility enables the system to be readily adapted to a variety of biological preparations. As a proof of concept, the authors employ the device to record blood flow velocities in cortical microcapillaries, arterioles, and venules, thereby directly visualizing vasodilatation and vasoconstriction dynamics and permitting quantitative analysis of neurovascular coupling across cortical layers in awake mice.

The authors demonstrate that the tunability of the Bessel beam can be exploited to match the numerical aperture to the vessel type: a high NA configuration, albeit slower scan, is optimal for resolving flow in capillaries, whereas a low NA setting provides faster acquisition suitable for arterioles and venules. By implementing a one-dimensional line scan with the Bessel beam, they achieve an imaging speed that is twentyfold faster than conventional frame-by-frame scanning, which proves sufficient to capture hemodynamic transients before and after an induced ischemic stroke.

In addition to pure observation, the authors integrate a co-propagating Gaussian line to the system, allowing simultaneous imaging and photostimulation within the same focal plane. This capability addresses a common limitation of other Bessel beam implementations, in which the observation and perturbation planes often become misaligned when the Bessel beam is altered. The manuscript also emphasizes the advantage of Bessel beam excitation for calcium imaging after a perturbation, because it captures neuronal activity in planes both above and below the nominal focal plane, signals that would be missed with a standard Gaussian focus. Finally, the authors apply the technique to investigate the neuroimmune response following targeted microglial ablation; they report that adjacent microglia extend processes toward the injury site while retracting processes in the opposite direction.

Overall, the work offers a technically straightforward yet powerful extension to existing two-photon platforms, providing high-speed, volumetric imaging and stimulation capabilities that are well-suited to a broad range of neurovascular and neuroimmune studies. The experimental validation is quite thorough, and the presented data convincingly illustrates the benefits of the approach.

Strengths:

The authors present a truly clever and inexpensive optical module that can be integrated into almost any two-photon microscope, providing a tunable Bessel beam with a minimal modification of the existing system. The experimental data and accompanying quantitative analysis convincingly demonstrate that the system can reveal physiological events, such as capillary flow, calcium transients across multiple axial planes, and microglial process dynamics, that are difficult or impossible to capture with a conventional Gaussian beam. The breadth of experiments chosen for the manuscript illustrates the practical utility of the device and supports the authors' conclusions that it extends the functional repertoire of standard two-photon microscopy.

We sincerely thank the reviewer for the thoughtful and encouraging feedback. We're glad that the technical design and broad applicability of the tBessel module came through clearly, and we appreciate the recognition of its ease of integration and ability to capture dynamic physiological processes.

Weaknesses:

The manuscript would benefit from a more detailed contextualisation of the claimed speed advantage. Although the authors mention other techniques in the introduction, they do not provide any direct comparison with other state-of-the-art high-speed two-photon approaches such as light beads microscopy (Demas et al., Nat. Methods 2021), temporal multiplexing schemes (Weisenburger et al., Cell 2019), or random access microscopy (Villette et al., Cell 2019). A brief comparison of imaging speed, spatial resolution, and instrumental complexity would enable readers to assess the relative merits of the present method.

We thank the reviewer for this important suggestion. We agree that a more explicit comparison with other high-speed two-photon imaging methods helps clarify the speed advantages of our system. Several existing approaches, including light-beads microscopy (LBM), temporal multiplexing, and AOD-based random-access microscopy, have demonstrated impressive high-speed volumetric imaging capabilities. Light-beads microscopy (Demas et al., 2021) reported imaging over a large volume of 5.4 × 6 × 0.5 mm^3^ at 2 Hz. However, this large-volume acquisition used 5-μm lateral pixel sampling, corresponding to an effective lateral resolution of approximately 10 μm. In a more comparable mesoscopic volume, LBM imaged 0.6 × 0.6 × 0.5 mm^3^ at 9.6 Hz with 1-μm lateral pixel sampling. In addition, the LBM module uses off-axis reflective concave mirrors, which require careful alignment, and the axial sampling range is not readily tunable. Temporal multiplexing approaches (Weisenburger et al., 2019), reported imaging over approximately 1 × 1 × 0.6 mm^3^ at 17 Hz. However, this volume rate was achieved with relatively coarse spatial resolution of approximately 5 μm, together with a more complex optical design involving multiplexed excitation, detection, and synchronization. AOD-based random-access microscopy (Nadella et al., 2016; Villette et al., 2019) provides very fast point or region sampling, and reported 250 × 250 μm^2^ imaging with 512 × 512 pixels and a 50-ns pixel dwell time, corresponding to ~0.5-μm pixel sampling and ~76 frames/s for two-dimensional imaging. However, volumetric imaging requires additional axial sampling, which lowers the effective 3D acquisition rate. In addition, AOD-based systems rely on diffractive beam steering, which introduces light loss due to finite diffraction efficiency and increases optical and calibration complexity. In comparison, tBessel-TPFM imaged a 0.4 × 0.4 × 0.12 mm^3^ volume at 58 Hz with 0.2-μm lateral pixel sampling. Our largest demonstrated imaging volume reached 2.5 × 2.5 × 0.45 mm^3^ while maintaining diffraction-limited lateral resolution. Therefore, compared with these high-speed volumetric approaches, tBessel-TPFM provides a distinct balance of volume rate and spatial sampling, and easier implementation simplicity.

A second limitation that warrants discussion is the inherent trade off between volumetric coverage and image specificity. Because the Bessel beam excites fluorescence throughout an extended axial range, the detector inevitably integrates signal from a three dimensional volume into a two dimensional image. In densely labelled tissue, this can lead to significant signal crosstalk, reducing contrast and complicating quantitative interpretation. A brief analysis of how labeling density affects the fidelity of flow or calcium measurements, or suggestions for mitigating crosstalk (e.g., computational deconvolution, adaptive excitation shaping, or combinatorial sparse labeling), would broaden the applicability of the technique.

We thank the reviewer for highlighting this important trade-off between volumetric coverage and image specificity in Bessel beam imaging. As Bessel beams project fluorescence from multiple features along the z-axis onto the same x–y plane, longer beams expand depth coverage at the same acquisition speed but can confound signals from axially spaced structures (Line 119-121 in manuscript). For densely labeled samples, the probability of having structures overlap in their x-y locations is high, and thus a shorter beam should be used. In sparsely labeled samples, structures have a lower probability of overlapping, and thus longer foci can be used (Line 166-168 in manuscript). Additionally, at the same NA, longer Bessel beam have more energy in the side rings surrounding the central peak, which may lead to higher background signal (Line 121-123 in manuscript) (Lu et al., 2017). These reasons necessitate to have not only NA tuning, but also independent length tuning (ΔNA tuning) to optimize imaging Bessel length to provide a balance between structural overlap that obscures signal localization, and the volumetric speedup, in any given sample based on labeling density and imaging goals, which are realized in our tBessel design.

Reference:

Demas, J., Manley, J., Tejera, F., Barber, K., Kim, H., Traub, F.M., Chen, B., Vaziri, A., 2021. High-speed, cortex-wide volumetric recording of neuroactivity at cellular resolution using light beads microscopy. Nat Methods 18, 1103–1111. https://doi.org/10.1038/s41592-021-01239-8

Göbel, W., Helmchen, F., 2007. In Vivo Calcium Imaging of Neural Network Function. Physiology 22, 358–365. https://doi.org/10.1152/physiol.00032.2007

Grewe, B.F., Voigt, F.F., van ’t Hoff, M., Helmchen, F., 2011. Fast two-layer two-photon imaging of neuronal cell populations using an electrically tunable lens. Biomed Opt Express 2, 2035–2046. https://doi.org/10.1364/BOE.2.002035

Huang, C., Tai, C.-Y., Yang, K.-P., Chang, W.-K., Hsu, K.-J., Hsiao, C.-C., Wu, S.-C., Lin, Y.-Y., Chiang, A.-S., Chu, S.-W., 2019. All-Optical Volumetric Physiology for Connectomics in Dense Neuronal Structures. iScience 22, 133–146. https://doi.org/10.1016/j.isci.2019.11.011

Lu, R., Sun, W., Liang, Y., Kerlin, A., Bierfeld, J., Seelig, J.D., Wilson, D.E., Scholl, B., Mohar, B., Tanimoto, M., Koyama, M., Fitzpatrick, D., Orger, M.B., Ji, N., 2017. Video-rate volumetric functional imaging of the brain at synaptic resolution. Nat Neurosci 20, 620–628. https://doi.org/10.1038/nn.4516

Nadella, K.M.N.S., Roš, H., Baragli, C., Griffiths, V.A., Konstantinou, G., Koimtzis, T., Evans, G.J., Kirkby, P.A., Silver, R.A., 2016. Random-access scanning microscopy for 3D imaging in awake behaving animals. Nat Methods 13, 1001–1004. https://doi.org/10.1038/nmeth.4033

Podgorski, K., Ranganathan, G., 2016. Brain heating induced by near-infrared lasers during multiphoton microscopy. Journal of Neurophysiology 116, 1012–1023. https://doi.org/10.1152/jn.00275.2016

Sofroniew, N.J., Flickinger, D., King, J., Svoboda, K., 2016. A large field of view two-photon mesoscope with subcellular resolution for in vivo imaging [WWW Document]. eLife. https://doi.org/10.7554/eLife.14472

Villette, V., Chavarha, M., Dimov, I.K., Bradley, J., Pradhan, L., Mathieu, B., Evans, S.W., Chamberland, S., Shi, D., Yang, R., Kim, B.B., Ayon, A., Jalil, A., St-Pierre, F., Schnitzer, M.J., Bi, G., Toth, K., Ding, J., Dieudonné, S., Lin, M.Z., 2019. Ultrafast Two-Photon Imaging of a High-Gain Voltage Indicator in Awake Behaving Mice. Cell 179, 1590-1608.e23. https://doi.org/10.1016/j.cell.2019.11.004

Weisenburger, S., Tejera, F., Demas, J., Chen, B., Manley, J., Sparks, F.T., Traub, F.M., Daigle, T., Zeng, H., Losonczy, A., Vaziri, A., 2019. Volumetric Ca2+ Imaging in the Mouse Brain Using Hybrid Multiplexed Sculpted Light Microscopy. Cell 177, 1050-1066.e14. https://doi.org/10.1016/j.cell.2019.03.011

Yang, W., Carrillo-Reid, L., Bando, Y., Peterka, D.S., Yuste, R., 2018. Simultaneous two-photon imaging and two-photon optogenetics of cortical circuits in three dimensions. eLife 7, e32671. https://doi.org/10.7554/eLife.32671
